# Effects of Mulberry Leaf Extract on the Growth Performance, Organ Indices, Flesh Quality, Collagen Metabolism, and Myofiber Development of Grass Carp (*Ctenopharyngodon idella*)

**DOI:** 10.3390/ijms27156904

**Published:** 2026-08-01

**Authors:** Yan Lin, Qi’ao Song, Xizheng Sun, Wenqiang Jiang, Siyue Lu, Zhengyan Gu, Linghong Miao

**Affiliations:** 1 Key Laboratory of Freshwater Fisheries and Germplasm Resources Utilization, Ministry of Agriculture and Rural Affairs, Freshwater Fisheries Research Center, Chinese Academy of Fishery Sciences, Wuxi 214081, China; liny@ffrc.cn (Y.L.);; 2 College of Fisheries and Life Science, Dalian Ocean University, Dalian 116023, China; 3 Wuxi Fisheries College, Nanjing Agricultural University, Wuxi 214081, China

**Keywords:** mulberry leaf extract, *Ctenopharyngodon idella*, flesh quality, collagen metabolism, myofiber development

## Abstract

To investigate the effects of mulberry leaf extract (MLE) on the growth performance, organ indices, and flesh quality of grass carp (*Ctenopharyngodon idella*), a basal diet (Control, Crude protein 30.25%, Crude lipid 7.23%) and three MLE-supplemented diets containing 1%, 2%, and 4% MLE (MLE1%, MLE2%, MLE4%) were formulated. A total of 160 grass carp (259.7 ± 1.1 g) were randomly allocated into 16 cages (1 m × 1 m × 1.2 m), with 10 fish per cage and 4 replicate cages per treatment group. Throughout the 8-week feeding trial, juvenile grass carp were fed three times daily (08:00, 12:00, and 17:00) at a daily feeding rate of 1.5–2.0% body weight. At the end of the trial, growth performance was assessed. Additionally, four fish were randomly sampled from each cage to determine the organ indices, flesh quality parameters, and gene expression levels. In the results, the final average body weight of all groups ranged from 909.00 to 942.97 g, and all the groups exhibited no significant difference in growth performance (*p* > 0.05). MLE-supplemented groups had significantly decreased visceral somatic index and hepatic somatic index (*p* < 0.05). In terms of flesh quality, all MLE-supplemented groups increased the flesh shear force, springiness, whiteness, and lightness (*p* < 0.05). Compared with the Control group, the MLE4% group had increased flesh hardness, gumminess, and chewiness, from 2899.52 g, 1310.62, and 516.59 to 4122.99 g, 1958.48, and 887.76, respectively (*p* < 0.05). MLE significantly decreased the 48 h thawing loss (MLE2%), 96 h thawing loss (MLE1%, MLE2%, MLE4%), and flesh crude lipid content (MLE2%, MLE4%) (*p* < 0.05). In terms of collagen metabolism, compared with the Control group, MLE significantly increased the flesh collagen content (MLE2% and MLE4%) (*p* < 0.05), upregulated the expression level of La ribonucleoprotein 6 (MLE4%) and tissue inhibitor of metalloproteinase 1 (MLE4%). Matrix metallopeptidase 2 was significantly downregulated in all MLE-supplemented groups (*p* < 0.05). In terms of myofiber development, compared with the Control group, the myofiber density was higher, and the myofiber diameter was lower in all MLE-supplemented groups (*p* < 0.05). MLE significantly upregulated the expression level of mammalian target of rapamycin (MLE4%), musculoskeletal embryonic nuclear protein 1 (MLE4%), myogenic differentiation 1 (MLE2%, MLE4%), and myogenic factor 5 (MLE1%, MLE2%, MLE4%) (*p* < 0.05). Myotubularin-related protein 8 was significantly downregulated in all MLE–supplemented groups (*p* < 0.05). In conclusion, dietary supplementation with 2–4% MLE improves the flesh textural properties, chroma and water-holding capacity of grass carp. MLE improves the flesh quality of grass carp by enhancing collagen deposition and myofiber development.

## 1. Introduction

Aquatic products have garnered heightened consumer interest due to their distinct sensory attributes and high nutritional value. From 1961 to 2022, the global annual per capita apparent consumption of aquatic products grew from 9.1 kg to 20.7 kg, exceeding the growth rate of meat products produced by terrestrial animals [1]. As living standards continue to rise, consumer expectations regarding the freshness, texture, and overall quality of aquatic products have also increased. Consequently, research focusing on quality evaluation and the underlying biological mechanisms governing quality formation in aquatic products has attracted growing attention [2,3,4]. With the development of aquaculture, researchers are paying more attention on the relationship between nutritional regulation and product quality. It has been demonstrated that modulating flesh flavor and quality through dietary interventions, particularly via novel feed ingredients and functional additives such as plant extracts, represents a practical and sustainable technical strategy [5,6,7].

Mulberry leaves (ML) are recognized as a high-quality forage plant resource that is rich in polysaccharides, flavonoids, alkaloids, and other bioactive compounds [8]. They exhibit multiple physiological functions, including antioxidant, hypoglycemic, and lipid metabolism regulatory properties [9,10,11]. In recent years, mulberry leaf powder (MLP), mulberry leaf extracts (MLE) and their bioactive components have been increasingly explored as novel functional feed ingredients in aquaculture. Studies on various fish species such as blunt snout bream (*Megalobrama amblycephala*) [12,13,14], largemouth bass (*Micropterus salmoides*) [15], tilapia (*Oreochromis niloticus*) [16], spotted sea bass (*Lateolabrax maculatus*) [17,18], African catfish (*Clarias gariepinus*) [19], and Chinese perch (*Siniperca chuatsi*) [20] have demonstrated their potential to enhance growth, antioxidant capacity, disease resistance, and lipid metabolism. In terrestrial animals, including pigs [21,22], chickens [23,24], rabbits [25], and lambs [26], MLP, MLE, and their active components have been shown to improve muscle chroma, enhance water-holding capacity and shear force, promote protein metabolism and myofiber development, thereby positively influencing meat quality. However, studies addressing the effects of MLP, MLE, and their bioactive components on fish muscle quality remain scarce, which substantially limits the application potential of mulberry resources in aquaculture. Our previous studies demonstrated that MLP, MLE, and mulberry leaf polysaccharides promoted lipolysis in fish, reduced lipid deposition in muscle, and increased fillet hardness, yet the regulatory effects on muscle texture formation and nutritional quality remain insufficiently characterized [12,14,27].

Fish muscle is primarily composed of muscle fibers and connective tissue. Studies have shown that the textural properties of fish muscle are closely associated with the structural characteristics of the muscle’s connective tissue and the architectural features of the muscle fibers [28,29,30,31]. Collagen is the main protein in connective tissue. Its synthesis and deposition are regulated by genes related to collagen metabolism, and it plays a significant role in influencing the texture and nutritional value of muscle [28,32,33,34,35,36]. Plant-derived additives, such as Eucommia ulmoides extract and rutin, can improve muscle quality in grass carp (*Ctenopharyngodon idella*) by modulating collagen metabolism [37,38]. In addition to collagen metabolism, the myofiber characteristics, particularly fiber diameter and density, are regulated by genes related to myofiber development and are key determinants of muscle texture [28,39,40,41,42]. Studies on grass carp have further confirmed that regulation of myofiber development significantly influences muscle texture traits, such as hardness and chewiness [30,43]. Therefore, investigating collagen metabolism and myofiber development provides a mechanistic basis for elucidating the regulatory effects of MLE on grass carp muscle quality.

Grass carp is one of the most important aquaculture species worldwide, with an annual production of approximately 6 million tons [1]. Currently, substantial research efforts have focused on the nutritional regulation and molecular mechanisms underlying muscle quality formation in grass carp [34,37,38,39,40]. However, it remains unclear whether, in grass carp, MLE can modulate muscle texture properties and the associated mechanisms involving collagen metabolism and myofiber development. Therefore, the present study aimed to systematically investigate the effects of MLE on muscle quality in grass carp and to analyze the underlying regulatory mechanisms involving collagen metabolism and myofiber development.

## 2. Results

### 2.1. Growth Performance and Organ Indexes of Grass Carp

As shown in Table 1, final body weight (FBW), weight gain rate (WGR), specific growth rate (SGR), and feed conversion ratio (FCR) did not differ significantly among the experimental groups (*p* > 0.05). However, all MLE supplementation groups (MLE1%, MLE2%, MLE4%) significantly reduced the visceral somatic index (VSI) and hepatic somatic index (HSI) compared with the Control group (*p* < 0.05).

### 2.2. Flesh Physical Characteristics and Proximate Composition of Grass Carp

As shown in Table 2, compared with the Control group, the MLE1%, MLE2%, and MLE4% groups showed significantly higher flesh shear force, springiness, lightness (L*), and whiteness (*p* < 0.05), while hardness, gumminess, and chewiness were significantly higher in the MLE4% group (*p* < 0.05). Dietary supplementation with MLE significantly reduced the 48 h thawing loss in the MLE2% group and the 96 h thawing loss in the MLE1%, MLE2%, and MLE4% groups compared with the Control group (*p* < 0.05). In addition, the flesh crude lipid content in the MLE2% and MLE4% groups was significantly lower than that in the Control group (*p* < 0.05).

### 2.3. Flesh Collagen Metabolism of Grass Carp

**Figure 1:**
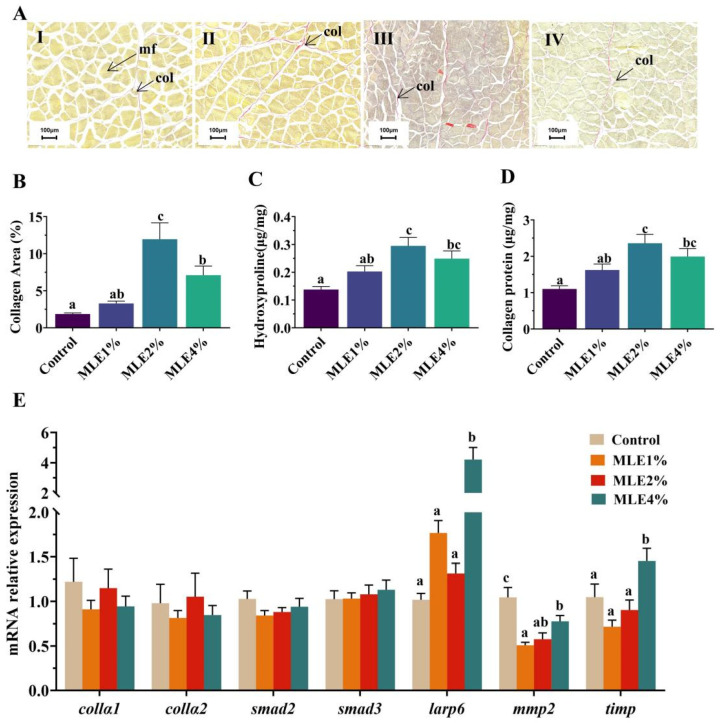
Effects of dietary mulberry leaf extract on flesh collagen metabolism of grass carp. (**A**) Muscle sirius red-staining results. (**B**) Proportion of muscle collagen area. (**C**) Muscle hydroxyproline content. (**D**) Muscle collagen content. (**E**) mRNA expression levels of genes related to muscle collagen metabolism. Different lowercase letters in the column indicate significant differences (*p* < 0.05). Values are presented as mean ± SME, *n* = 4. mf, myofiber; col, collagen. Collagen type I α 1 (*collα1*), collagen type I α 2 (*collα2*), SMAD family member 2 (*smad2*), SMAD family member 3 (*smad*3), La ribonucleoprotein 6 (*larp6*), matrix metallopeptidase 2 (*mmp2*), tissue inhibitor of metalloproteinase 1 (*timp*).

The Sirius red-staining results indicated that collagen deposition in the MLE2% and MLE4% groups was significantly increased compared with the Control group. The contents of hydroxyproline and collagen were also significantly elevated (*p* < 0.05) (Figure 1A–D). The expression levels of La ribonucleoprotein 6 (*larp6*) and the tissue inhibitor of metalloproteinase 1 (*timp*) genes in the MLE4% group were significantly higher than those in other groups (*p* < 0.05). Compared with the Control group, matrix metallopeptidase 2 (*mmp2*) was significantly downregulated in the MLE1%, MLE2%, and MLE4% groups (*p* < 0.05). There were no significant differences in the expression levels of collagen type I α 1 (*collα1*), collagen type I α 2 (*collα2*), SMAD family member 2 (*smad2*), and SMAD family member 3 (*smad3*) genes among the different groups (*p* > 0.05) (Figure 1E).

### 2.4. Flesh Myofiber Development of Grass Carp

**Figure 2:**
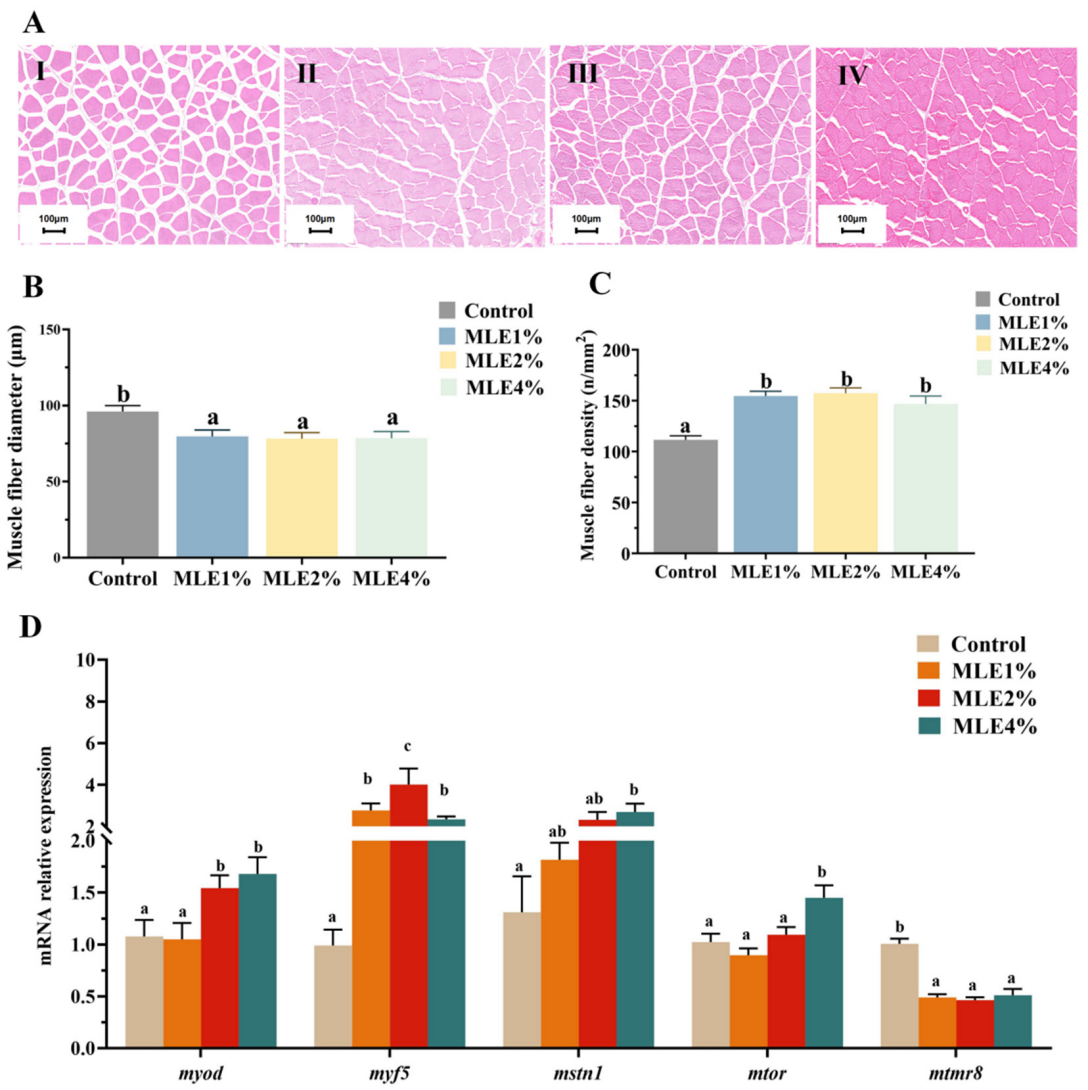
Effects of dietary mulberry leaf extract on myofiber development of grass carp. (**A**) Muscle H&E staining results. (**B**) Muscle fiber diameter. (**C**) Muscle fiber density. (**D**) mRNA expression levels of genes related to myofiber development. Different lowercase letters in the column indicate significant differences (Tukey’s test, *p* < 0.05). Values are presented as mean ± SME, *n* = 4. mf, myofiber. Myogenic differentiation 1 (*myod*), myogenic factor 5 (*myf5*), musculoskeletal embryonic nuclear protein 1 (*mstn1*), the mechanistic target of rapamycin (*mtor*), myotubularin-related protein 8 (*mtmr8*).

H&E staining results showed that the myofiber diameter was significantly decreased, whereas the myofiber density was significantly increased in the MLE1%, MLE2%, and MLE4% groups compared with the Control group (*p* < 0.05) (Figure 2A–C). The expression of myogenic differentiation 1 (*myod*) was higher in the MLE2% and MLE4% groups than in the Control and MLE1% groups (*p* < 0.05). Additionally, the expression of myogenic factor 5 (*myf5*) was significantly increased, whereas the expression of myotubularin-related protein 8 (*mtmr8*) was significantly decreased in the MLE1%, MLE2%, and MLE4% groups compared with the Control group (*p* < 0.05). Furthermore, the MLE4% group significantly elevated the expression levels of musculoskeletal embryonic nuclear protein 1 (*mstn1*) and the mechanistic target of rapamycin (*mtor*) compared with the Control group (*p* < 0.05) (Figure 2D).

### 2.5. Correlation Analysis

**Figure 3:**
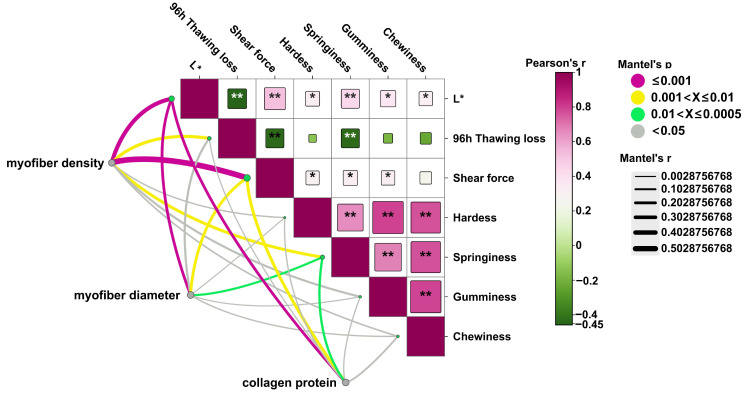
Correlations between myofiber density and diameter, collagen content, muscle color, thawing loss, and texture characteristics. The color gradient and size of the blocks reflect the magnitude of correlation derived from Pearson analysis. Pearson’s r denotes the strength of the linear relationship between variables, with red representing a positive correlation and green indicating a negative correlation. “*” denotes a significant correlation (*p* < 0.05), while “**” indicates a highly significant correlation (*p* < 0.01). Mantel test is applied to assess the relationships among muscle fiber diameter, muscle fiber density, collagen content, muscle color, thawing loss, and texture characteristics. Mantel’s *p* represents the significance, indicated by the color of the connecting lines; Mantel’s r indicates the strength of the correlation. The thicker the connection line, the stronger the correlation.

To investigate the effects of myofiber diameter and density, collagen content, flesh chroma, thawing loss, and texture characteristics of grass carp, a systematic analysis of the correlations among them was conducted using the Mantel test. As shown in Figure 3, the Pearson analysis indicated that the 96 h thawing loss was significantly negatively correlated with L*, shear force, and springiness (*p* < 0.01). There was a significant positive correlation among hardness, springiness, gumminess, and chewiness (*p* < 0.01). Shear force was significantly positively correlated with hardness, springiness, gumminess, and chewiness (*p* < 0.05). L* was significantly positively correlated with shear force and springiness (*p* < 0.01) and was also positively correlated with hardness, gumminess, and chewiness (*p* < 0.05). Myofiber density was significantly and closely related to L*, 96 h thawing loss, shear force, and springiness (Mantel’s *p* < 0.01); the myofiber diameter was significantly and closely related to L*, shear force, and springiness (Mantel’s *p* < 0.05). The collagen content was significantly and closely related to L*, shear force, and springiness (Mantel’s *p* < 0.05).

## 3. Discussion

### 3.1. Effects of Mulberry Leaf Extract on the Growth Performance and Organ Indices of Grass Carp

MLE is rich in bioactive components, including flavonoids, alkaloids, and polysaccharides [44], and has been increasingly applied as a functional feed ingredient in livestock, poultry, and aquatic animals [8,18]. In this study, MLE supplementation did not significantly affect growth performance or feed utilization, indicating that 1–4% inclusion levels of MLE had no adverse effects on growth in grass carp. Consistent results have been reported in largemouth bass (*Micropterus salmoides*), where supplementation with 0.05–1.0% MLE in high-starch diets did not significantly influence growth performance [45]. These results collectively suggest that MLE exhibits good nutritional safety and tolerance across a wide range of inclusion levels. In contrast, other studies have shown that lower dietary levels of MLE (0.05–0.9%) improved the growth performance and feed utilization rate of spotted sea bass (*Lateolabrax maculatus*), Chinese giant salamanders (*Andrias davidianus*), and mandarin fish (*Siniperca chuatsi*) [17,20,46]. Such discrepancies may be attributed to species-specific metabolic characteristics, diet composition, and dosage-dependent effects of MLE. Supplementation with 0.20–1.0% MLE in high-carbohydrate diets significantly reduced the HSI of *Micropterus salmoides*, and 1.0% MLE significantly reduced the VSI [45]. Consistent with these findings, the present study showed that all MLE-supplemented groups exhibited significantly lower VSI and HSI. In addition, dietary MLE supplementation decreased the muscle crude lipid content. Similar lipid-lowering effects of MLE have been observed in Chinese giant salamanders (*Andrias davidianus*), where increasing dietary MLE levels led to an initial decrease in muscle lipid content, with the 0.6% and 0.9% MLE groups showing significantly lower values than the Control group [46]. Moreover, studies in pigs have demonstrated that MLP significantly inhibited backfat deposition, which may be attributed to the suppression of lipid synthesis pathways [47]. Collectively, the reduction in VSI and HSI observed in this study suggests that MLE may effectively alleviate excessive lipid accumulation in visceral tissues and the liver, which is likely associated with its regulatory effects on glucose and lipid metabolism [14,20]. This metabolic modulation may be partly attributable to the bioactive compounds present in MLE, particularly mulberry leaf polysaccharides and flavonoids, which have previously been reported to promote lipid catabolism, inhibit lipid synthesis, and reduce lipid deposition in animal tissues [48].

### 3.2. Effects of Mulberry Leaf Extract on the Flesh Physical Characteristics and Proximate Composition of Grass Carp

In the present study, 1–4% MLE increased muscle shear force and springiness, whereas MLE4% increased hardness, gumminess, and chewiness. The shear force, springiness, hardness, gumminess, and chewiness represent the texture properties of the muscle [49,50]. Studies on pigs have also shown that 12% MLP significantly enhances the shear force of the longissimus dorsi muscle [51]. Previous studies have shown that crisp grass carp fed broad beans also exhibited changes in texture properties, with higher hardness, springiness, and gumminess, resulting in improved flesh texture [50]. Therefore, MLE can improve the texture properties of grass carp muscle. Thawing loss can significantly reduce the muscle quality of aquatic animals, with the main losses including weight loss, juice leakage, and protein denaturation [52]. This is of great significance for subsequent freezing, storage, and processing of grass carp. The present study showed that the reduction in the 48 h and 96 h thawing loss during freezing indicates that MLE significantly enhances the water-holding capacity of grass carp and reduces water loss during the freezing process [53]. Muscle water-holding capacity is also reflected by cooking loss and drip loss. Previous studies have shown that 15% MLE significantly reduced cooking loss and drip losses in pigs [21]. These findings suggest that MLE has a positive effect on the water-holding capacity of muscles. The muscle of cyprinid fish is mainly white. High-quality fish flesh is white and lustrous [54]. L* and whiteness represent the chromaticity information of the muscle. In the present study, 1–4% MLE increased muscle L* and whiteness, thereby improving the appearance characteristics of the muscles. The L* of Tibetan pig muscle also increased in the group with 8% MLP supplementation [47].

In this study, indicators representing the texture properties of muscles, including shear force, hardness, springiness, gumminess, and chewiness, showed positive correlations. Moreover, L* was significantly positively correlated with these texture properties. However, the 96 h thawing loss was significantly negatively correlated with L* and these texture properties. These improvements in muscle texture, color characteristics, and water-holding capacity may be associated with alterations in collagen metabolism and myofiber development, both of which contribute to muscle structural properties [4,55,56,57].

### 3.3. Effects of Mulberry Leaf Extract on Flesh Collagen Metabolism of Grass Carp

The structural characteristics of the extracellular matrix of fish muscle cells are mainly reflected in the composition and deposition of collagen [58]. The connective tissue in muscle is mainly collagen, and collagen content is a significant correlation between the texture properties of muscle and the content of collagen in muscle [59]. In this study, the collagen deposition in the MLE2% and MLE4% groups was significantly increased. Studies on tilapia also showed that the addition of 500 mg/kg of mulberry leaf brass significantly increased the muscle collagen content [60]. Studies on grass carp have shown that muscle collagen is regulated by collagen synthesis-related genes such as *collα1*, *collα2*, *smads*, and the translation modification gene *larp6*, while *timp* can inhibit the degradation of collagen [34,39]. However, the expression levels of genes related to collagen synthesis (*collα1*, *collα2*, *smad2* and *smad3*) did not increase significantly in this study. MMPs are the main collagen-degrading enzymes, and their activity is inhibited by TIMPs. MMPs belong to the endopeptidase family and are involved in the degradation of the extracellular matrix in both physiological and pathological conditions [36]. LARP6 plays an important role in the initiation of collagen translation [30]. In this study, *mmp2* expression levels were significantly downregulated in the MLE1%, MLE2%, and MLE4% groups, whereas the MLE4% group upregulated the *larp6* and *timp* genes. These results indicate that MLE may promote collagen deposition by enhancing collagen modification and inhibiting collagen degradation. Increased collagen deposition facilitates the formation of collagen fibers through intermolecular cross-linking, thereby enhancing the structural stability of muscle tissue. Studies have found that pyridinoline cross-linking is a key factor influencing the hardness of the muscles [61]. The Mantel test results showed that the muscle collagen content was closely related to the shear force and springiness of the fish muscle. Collagen deposition has been proven to enhance the texture properties, including the muscle hardness and springiness of grass carp, and improve muscle quality [28,34]. These findings indicate that MLE increased the muscle collagen content of grass carp, which is beneficial for improving the muscle texture properties.

### 3.4. Effects of Mulberry Leaf Extract on the Flesh Myofiber Development of Grass Carp

This study found that the addition of 1–4%MLE significantly reduced the diameter of grass carp muscle fibers and significantly increased the density of muscle fibers. Studies have also shown that 8% MLE significantly reduced the myofiber diameter in pigs and significantly increased the muscle fiber density [22]. Studies on grass carp have shown that upregulation of *myod*, *myf5,* and *mtor* can promote the development of muscle fibers [39,40,62,63]. Mainly expressed in the early stage of the myogenesis process, *myod* and *myf5* are involved in the generation and maintenance of myoblasts [28]. In our study, MLE upregulated the expression levels of *myod*, *myf5,* and *mtor*. Studies have shown that *mtor* activation can promote muscle fiber growth [42]. Studies in chickens have shown that copper derived from mulberry leaves significantly upregulated the mRNA expression of *myf5* in skeletal muscles during both the embryonic and growth stages, and the expression of *mtor* in the breast muscles of the offspring chicks increased [24]. The increase in the myofiber density of grass carp may be attributed to the upregulation of *myod* and *myf5* mRNA levels [64]. Meanwhile, *mtmr8*, which is involved in muscle fiber development, was significantly reduced at the 1–4% MLE addition level, indicating that the MLE can promote myofiber growth [41]. Inhibition of mstn1 promotes myofiber hypertrophy [65]. In the present study, the expression level of *mstn1* gradually increased with an increase in the dietary MLE supplement amount. This may be related to the reduction in myofiber diameter. Therefore, through the coordinated regulation of *myod*, *myf5*, *mtor,* and *mtmr8*, MLE promotes myofiber development, increases myofiber density, and reduces myofiber diameter.

Studies have shown that there is a significant correlation between myofiber characteristics and muscle texture properties. Smaller myofiber diameter and higher myofiber density contribute to greater muscle firmness and improved hardness and chewiness [66,67]. The Mantel test results in this study showed that the myofiber density was significantly and closely related to L*, 96-h thawing loss, shear force, and springiness, whereas myofiber diameter was significantly and closely related to L*, shear force, and springiness. These findings indicate that MLE causes changes in the structure of myofibers, which, in turn, affects the texture properties, chroma, and water-holding capacity of the grass carp flesh.

## 4. Materials and Methods

All procedures involving animal subjects were performed in strict accordance with internationally recognized ethical standards and institutional regulations, with a focus on ensuring animal welfare, humane handling, and minimizing physiological stress throughout the experimental period. This study was approved by the Animal Care and Use Committee of the Chinese Academy of Fishery Sciences (Authorization No. 20240801002).

### 4.1. Experimental Diets

Fish meal, soybean meal, rapeseed meal, cottonseed protein, wheat flour, and soybean oil served as the primary protein sources, carbohydrates, and lipid sources, respectively, which were used to formulate four isonitrogenous and isolipid experimental diets (Table 3). MLE was provided by Hubei Manborui Biotechnology Co., Ltd. (Songzi, China) and contained 1.25% 1–deoxynojirimycin, 9.68% mulberry leaf total flavonoids, and 11.35% mulberry leaf polysaccharides. The diets included a Control (without additive, 30.25% crude protein, 7.23% crude lipid) and three experimental diets supplemented with MLE at 1%, 2%, and 4% (referred to as MLE1%, MLE2%, and MLE4%). During the production of the feed, each raw material was first crushed and passed through a 60-mesh sieve. Then, it was accurately weighed according to the formulation and thoroughly mixed. During the mixing process, soybean oil and an appropriate amount of water were slowly added. Subsequently, the feed was mechanically extruded into pellets at room temperature using a flat-mode pelletizer (Chen’s Gaole Machinery Factory, Nanming Street, Xinchang County, Shaoxing City, Zhejiang Province, China; rotational speed of 300–350 r/min) to form sinking pellets with a particle size of approximately 1.5 mm. The prepared feed was naturally air-dried and then placed in sealed bags and stored at –20 °C for future use. The crude protein and crude lipid contents of the diets were determined according to the AOAC method [68]. The crude protein content was determined by the Kjeldahl nitrogen determination method (N factor = 6.25), and the crude lipid content was determined by the Soxhlet extraction method.

### 4.2. Experimental Fish and Feeding Trial

Prior to the initiation of the experiment, grass carp were acclimated to the experimental environment by being temporarily reared in the cage (6 m × 4 m × 1.0 m) of the Nanquan farming base of FFRC, CAFS (31.43° N, 120.29° E) for a duration of 15 days. The grass carp were fed with the Control diet three times daily at 08:00, 12:00, and 17:00 during the acclimation period. Then, healthy juvenile grass carp (initial body weight 259.7 ± 1.1 g) were randomly distributed into 16 cages (1 m × 1 m × 1.2 m) in one pond, with 10 fish per cage, and the average weight of each fish in the cages was calculated based on the total weight of 10 fish. Each group had four repetitions. During the 8-week feeding trial, the juvenile grass carp were fed three times daily (08:00, 12:00, and 17:00) with a feed platform at the bottom of the cage. After 20–30 min of feeding, the feed platform was slowly lifted to observe whether the feed had been completely consumed. If any feed remained, it was removed, dried, and weighed. Daily feed of each cage was 1.5–2.0% of the total fish weight, adjusted every 3 days based on intake and a 1.5 feed coefficient to estimate gain. After 4 weeks, the feed was calculated from actual weight.

During the trial, fish were maintained under a natural photoperiod and ambient water temperature (27–33 °C). Water quality parameters were kept within the following ranges: dissolved oxygen ≥ 6.0 mg/L, ammonia nitrogen ≤ 0.1 mg/L, and pH 7.0–7.5. No deaths occurred during the feeding trial.

### 4.3. Sample Collection

**Figure 4:**
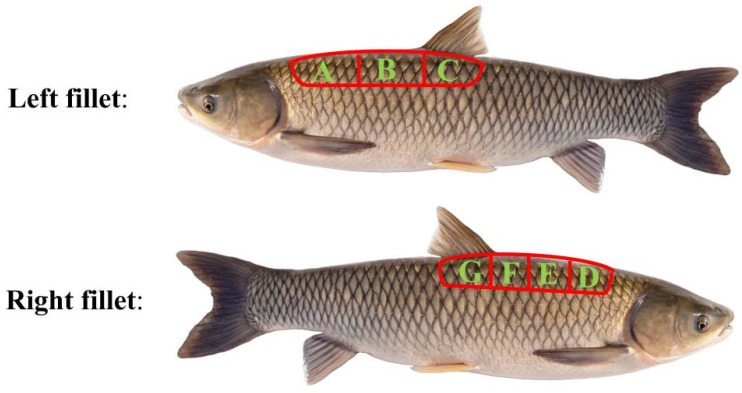
The sample site for flesh quality analyses. A, shear force; B, thawing loss; C, collagen content and proximate composition; D, texture profile analysis; E, color measurement; F, gene expression analysis; G, histological sectioning.

After an 8-week feeding trial, a 24-h fasting period was imposed. All fish were anesthetized (MS-222, 100 mg/L) to measure the FBW in each cage, and total feed intake and the number of fish per cage were counted to calculate WGR, SGR, and FCR. Subsequently, four fish were sampled from each cage. Body weight, visceral weight (with liver), and liver weight per fish were recorded to calculate VSI and HSI. Subsequently, the dorsal muscle per fish was dissected and divided into seven portions for different analyses as shown in Figure 4. The locations A and B of the left fillet were used for shear force analysis and thawing loss determination, respectively. Location C of the left fillet was stored at −20 °C for analysis of muscle collagen content and proximate composition. Locations D and E of the right fillet were used for texture profile analysis and chroma measurement, respectively. Location F of the right fillet was stored at −80 °C for gene expression analysis. Location G of the right fillet was fixed in 4% paraformaldehyde for histological sectioning.

### 4.4. Computational Formula

Weight gain rate (WGR, %), 100 × (FBW (g) − IBW (g))/IBW (g);

Specific growth rate (SGR, %/d), 100 × (ln FBW (g) − ln IBW (g))/days of feeding;

Feed conversion ratio (FCR ), Feed intake (g)/(FBW (g) − IBW (g));

Visceral somatic index (VSI, %), 100 × Final visceral weight (g)/FBW (g),

Hepatic somatic index (HSI, %), 100 × Final liver weight (g)/FBW (g).

Initial body weight: IBW (g), Final body weight: FBW (g).

### 4.5. Shear Force and Texture Profile Analysis

The dorsal muscle per fish was cut into two 1.0 cm × 1.0 cm × 1.0 cm blocks and tested for shear force and texture profile analysis (TPA), respectively. The TA.XT Plus texture analyzer (Stable Micro Systems, Godalming, UK) was used to analyse the shear force and TPA. The shear force probe model is A/CKB. The shear degree is 50%, and the test trigger force is 5 g. The pre-test, test, and post-test speeds are 2 mm/s, 1 mm/s, and 2 mm/s, respectively. For TPA testing, a cylindrical flat-bottom probe (P/50) was used for two compressions. The test speed is 2 mm/s, and there is a 3-s interval between the two compressions. The main detection indicators are hardness, springiness, gumminess, and chewiness. All texture parameters (hardness, springiness, gumminess, and chewiness) were automatically calculated using the Exponent software supplied with the texture analyzer according to the standard texture profile analysis (TPA) algorithm described by Bourne et al. [69].

### 4.6. Flesh Chroma and Thawing Loss

The dorsal muscle of each fish was cut into a 1.0 cm × 1.0 cm × 1.0 cm piece. Chroma parameters—lightness (L*), redness (a*), and yellowness (b*)—were measured using a high-precision colorimeter (NR200, Shenzhen City, China, Shenzhen Threenh Technology Co., Ltd.). Chroma analysis was conducted in a chamber with uniform lighting. According to the instrument operation manual, first perform whiteboard calibration by selecting the L* a* b* color space. Position the measurement port vertically against the flesh surface and press firmly to prevent light leakage. Then, read the values of L*, a*, and b*. Muscle whiteness was calculated according to the following formula: 100−(100−L*)^2+a*^2+b*^2.

The thawing loss was determined as follows. Dorsal muscle samples (approximately 5 g per fish) were accurately weighed and frozen at −20 °C. After freezing for 48 h and 96 h, the samples were thawed at 4 °C for 2 h. Following thawing, surface moisture was thoroughly absorbed with absorbent paper. The samples were then weighed again, and the thawing loss was calculated.

For 48 h thawing loss (%): (dorsal muscle weight before frozen—48 h dorsal muscle weight)/dorsal muscle weight before frozen × 100;

For 96 h thawing loss (%): (dorsal muscle weight before frozen—96 h dorsal muscle weight)/dorsal muscle weight before frozen × 100.

### 4.7. Proximate Composition and Collagen Content

The moisture, crude protein, and crude lipid of the dorsal muscle were determined according to the AOAC method [68]. The moisture content was calculated by drying the samples in a 105 °C oven until a constant weight was achieved; the crude protein content was determined by the Kjeldahl nitrogen determination method (N factor = 6.25), and the crude lipid content was determined by the Soxhlet extraction method. The collagen content kits (A030-2) were purchased from Nanjing Jiancheng Bioengineering Institute (Nanjing, China), and the measurements were performed according to the kit instructions.

### 4.8. HE Staining and Sirius Red Staining

After the dorsal muscles (four samples per cage, sixteen samples per group) were fixed with 4% paraformaldehyde for 24 h, the samples were routinely embedded in paraffin and sectioned. One part was stained with hematoxylin–eosin (HE), and the characteristics of myofibers were observed using an optical microscope (Nikon Eclipse E100, Nikon Corporation, Tokyo, Japan) and imaging system (Nikon DS-U3, Nikon Corporation, Tokyo, Japan). The diameter and density of the myofibers were analyzed using the Imagej/FIJI image-processing software [30]. Under microscopic examination, approximately 300–500 myofibers were observed per section. The fiber diameter obtained through cross-sectional area measurement was calculated using the formula: cross-sectional area = π × fiber radius^2^. Fiber density is calculated as the number of fibers per square millimeter of muscle cross-sectional area. The other part was stained with sirius red, and the deposition of collagen fibers was observed using an optical microscope (Nikon Eclipse E100) and imaging system (Nikon DS-U3). The imaging system was used to capture images (Nikon DS-U3). The Imagej/FIJI image processing software (2.0) was used to calculate the percentage of the area occupied by collagen fibers.

### 4.9. Quantitative Real-Time PCR

The RNA extraction method, reverse transcription method, and fluorescence quantitative method were referred to our previous research [70]. The total RNA of the dorsal muscle was extracted using Trizol reagent, chloroform, isopropanol, and 75% ethanol, and the RNA precipitate was dissolved with DEPC water. The Total RNA concentration and quality were assessed using a Nano Drop^®^2000 spectrophotometer (Thermo Fisher Scientific, Waltham City, MA, USA). A PrimeScript™ RT reagent Kit with gDNA Eraser (RR047A, Takara Bio Inc., Kusatsu, Japan) was used to reverse transcribe RNA to cDNA. Real-time quantitative polymerase chain reaction was carried out by using a CFX96TM Real-time PCR detection system (BIO-RAD, Hercules, CA, USA). TB Green^®^ Premix Ex Taq™ II (RR820A, Takara Bio Inc., Kusatsu, Japan) was used to detect target gene expression levels (39 cycles: 95 °C for 3 min, 95 °C for 10 s, and 55 °C for 30 s). *β-actin* and *ef-1α* were used as reference genes, and the gene expression levels were calculated by the 2^-ΔΔct^ method. Specific primers of *collα1*, *collα2*, *smad2*, *smad3*, *larp6*, *mmp2*, *timp*, *myod*, *myf5*, *mstn1*, *mtor*, and *mtmr8* were designed by NCBI and synthesized by Shanghai Biotechnology Company, Shanghai, China (Table 4).

### 4.10. Correlation Analysis

The correlations among myofiber density, myofiber diameter, collagen content, muscle color, thawing loss, and texture characteristics were analyzed using the ChiPlot online platform https://www.chiplot.online/ (accessed on 21 January 2026). Pearson’s r was used to denote the strength of the linear relationship between variables. The Mantel test was applied to assess the relationships among muscle fiber diameter, muscle fiber density, collagen content, muscle color, thawing loss, and texture characteristics.

### 4.11. Statistical Analysis

The normality of data distribution and the homogeneity of variances were assessed using the Kolmogorov–Smirnov test and Levene’s test, respectively, in SPSS (version 20.0). Statistical differences among treatments were determined by one-way analysis of variance (ANOVA), followed by Tukey’s multiple comparison test, and *p* < 0.05 was considered statistically significant. Data are presented as the mean ± standard error of the means.

## 5. Conclusions

In summary, dietary supplementation with 2–4% mulberry leaf extract (MLE) improved the textural properties, chroma, and water-holding capacity of grass carp flesh. These improvements are associated with a coordinated regulation of collagen metabolism and myofiber development. Specifically, MLE promoted collagen deposition by enhancing collagen modification and suppressing collagen degradation, thereby stabilizing the muscle connective tissue structure. Concurrently, MLE reshaped the myofiber architecture by promoting myofiber development, inhibiting myofiber hypertrophy, and increasing fiber density, which collectively contributed to superior flesh quality. Future studies should further elucidate the causal molecular mechanisms and key bioactive components underlying the MLE-mediated regulation of collagen metabolism and myofiber remodeling, as well as validate its effects across different growth stages and production conditions.

## Figures and Tables

**Table 1:** Effects of mulberry leaf extract on growth performance and organ indices of grass carp.

Items	Control	MLE1%	MLE2%	MLE4%
FBW	942.97 ± 37.60 ^a^	910.02 ± 26.20 ^a^	909.00 ± 38.19 ^a^	929.17 ± 29.59 ^a^
WGR	264.21 ± 14.37 ^a^	253.0 4 ± 6.33 ^a^	241.02 ± 13.47 ^a^	258.69 ± 9.45 ^a^
SGR	2.08 ± 0.06 ^a^	2.04 ± 0.03 ^a^	1.97 ± 0.06 ^a^	2.06 ± 0.04 ^a^
FCR	1.85 ± 0.14 ^a^	2.11 ± 0.08 ^a^	2.13 ± 0.14 ^a^	1.97 ± 0.07 ^a^
VSI	8.20 ± 0.20 ^b^	6.95 ± 0.16 ^a^	7.23 ± 0.26 ^a^	7.01 ± 0.28 ^a^
HSI	2.07 ± 0.08 ^b^	1.50 ± 0.06 ^a^	1.58 ± 0.10 ^a^	1.64 ± 0.11 ^a^

Notes: Different lowercase superscripts in the same row indicate significant differences based on one-way ANOVA followed by Tukey’s test (*p* < 0.05). Values are presented as mean ± SME, *n* = 4.

**Table 2:** Effects of dietary mulberry leaf extract on flesh physical characteristics and proximate composition of grass carp.

Items	Control	MLE1%	MLE2%	MLE4%
Texture characteristics				
Shear force (N/s)	3.04 ± 0.24 ^a^	5.07 ± 0.23 ^b^	5.10 ± 0.24 ^b^	5.60 ± 0.12 ^b^
Hardness (g)	2899.52 ± 306.44 ^a^	3249.48 ± 262.61 ^ab^	3118.14 ± 379.64 ^ab^	4122.99 ± 290.30 ^b^
Springiness (g)	0.38 ± 0.01 ^a^	0.44 ± 0.01 ^b^	0.44 ± 0.01 ^b^	0.45 ± 0.01 ^b^
Gumminess	1310.62 ± 158.32 ^a^	1545.23 ± 149.34 ^ab^	1652.18 ± 183.58 ^ab^	1958.48 ± 157.59 ^b^
Chewiness	516.59 ± 75.69 ^a^	683.94 ± 76.73 ^ab^	648.92 ± 97.18 ^ab^	887.76 ± 92.26 ^b^
Chroma				
Whiteness	36.18 ± 6.99 ^a^	58.17 ± 1.53 ^b^	63.06 ± 1.02 ^b^	64.23 ± 1.16 ^b^
L* (Lightness)	52.55 ± 0.95 ^a^	58.58 ± 1.58 ^b^	64.54 ± 1.16 ^c^	65.58 ± 1.19 ^c^
Water–holding capacity				
48 h Thawing loss, %	5.19 ± 0.39 ^b^	4.88 ± 0.27 ^b^	3.79 ± 0.17 ^a^	4.32 ± 0.22 ^ab^
96 h Thawing loss, %	7.29 ± 0.52 ^b^	5.85 ± 0.27 ^a^	5.02 ± 0.18 ^a^	5.47 ± 0.36 ^a^
Proximate components (wet basis)				
Moisture, %	76.01 ± 0.45 ^a^	76.93 ± 0.29 ^a^	77.12 ± 0.21 ^a^	77.00 ± 0.26 ^a^
Crude protein, %	19.84 ± 0.59 ^a^	19.72 ± 0.23 ^a^	19.86 ± 0.39 ^a^	20.14± 0.22 ^a^
Crude lipid, %	4.38 ± 0.36 ^b^	3.05 ± 0.41 ^ab^	2.32 ± 0.08 ^a^	2.77 ± 0.32 ^a^

Notes: Different lowercase superscripts in the same row indicate significant differences based on one-way ANOVA followed by Tukey’s test (*p* < 0.05). Values are presented as mean ± SME, *n* = 4.

**Table 3:** Feed formulation.

Ingredients, %	Control	MLE1%	MLE2%	MLE4%
Mulberry leaf extract	0.00	1.00	2.00	4.00
Fish meal ^a^	4.00	4.00	4.00	4.00
Soybean meal ^a^	26.00	26.00	26.00	26.00
Rapeseed meal ^a^	20.00	20.00	20.00	20.00
Cottonseed protein ^a^	9.00	9.00	9.00	9.00
Wheat flour ^b^	18.60	18.60	18.60	18.60
Rice bran ^a^	20.00	15.30	15.30	15.30
Soybean oil ^b^	0.00	0.70	0.70	0.70
Ca (H_2_PO_4_)_2_ ^a^	2.00	2.00	2.00	2.00
Mineral premix ^c^	0.30	0.30	0.30	0.30
Vitamin premix ^c^	0.10	0.10	0.10	0.10
Microcrystalline cellulose ^a^	0.00	3.00	2.00	0.00
Nutritional composition				
Crude protein	30.25	30.01	29.98	30.12
Crude lipid	7.23	7.16	7.30	7.28

Note: ^a^ Provided by Da Bei Nong Stock Co., Ltd. (Huai’an, China). ^b^ Purchased from Jingdong Mall; wheat flour is Wudeli brand flour, and soybean oil is Fulinmen first-class soybean oil. ^c^ Provided by Wuxi Hanove Animal Health Products Co., Ltd. (Wuxi, China); Vitamin premix (per kg content): vitamin A, 900,000 IU; vitamin D, 25,000 IU; vitamin E, 4500 mg; vitamin K3, 220 mg; vitamin B1, 320 mg; vitamin B2, 1090 mg; vitamin B5, 2000 mg; vitamin B6, 5000 mg; vitamin B12, 116 mg; pantothenic acid, 1000 mg; folic acid, 165 mg; choline, 60,000 mg; biotin, 50 mg; niacin, 2500 mg; Mineral premix (per kg content): calcium diphosphate, 20 g; sodium chloride, 2.6 g; potassium chloride, 5 g; magnesium sulfate, 2 g; ferrous sulfate, 0.9 g; zinc sulfate, 0.06 g; cupric sulfate, 0.02 g; manganese sulfate, 0.03 g; cobalt chloride, 0.05 g; potassium iodide, 0.004 g.

**Table 4:** Gene primers for RT-PCR.

Genes	GenBank Accession No.	Primer	Primer Sequences (5′-3′)	Amplification Efficiency	R^2^
*β-actin*	M25013	F	GGCTGTGCTGTCCCTGTA	106.10%	0.987
		R	GGGCATAACCCTCGTAGAT		
*ef1α*	GQ266394	F	CGCCAGTGTTGCCTTCGT	109.40%	0.986
		R	CGCTCAATCTTCCATCCCTT		
*collα1*	HM363526.1	F	CAACAGCCGCTTCACATACA	90.90%	0.993
		R	GGCGATGTCAATAATAGGCAG		
*collα2*	HM771241.1	F	CATTGGTGGCGCAGATCAAG	90.50%	0.990
		R	TCCTCTCCGATAGAGCCCAG		
*smad2*	DQ912858.1	F	GTCCTCCATCTTGCCTTTCAC	96.50%	0.986
		F	CTTCTCGCACCATTTCTCCTC		
*smad3*	DQ912859.1	R	ATTGAGCCTCCGAGCAACTAT	94.60%	0.989
		F	GAAAGATTTGGGGAACCTGTG		
*larp6*	OL438919	F	CTGAGGAGTGTGCCATCGTAG	106.40%	0.977
		R	TTCTTGGGAGGTTTGGTGCC		
*mmp2*	XM051898974	R	GAGCTGTGGACATTAGGAGAAG	105.32%	0.978
		F	GAACAAGAGCTCATGAGGACAG		
*timp*	HQ153832.1	R	GTGGTCCAGTGTTCCGTCAT	108.30%	0.984
		F	GCACCCAGTCAGTCCAAAGA		
*myod*	JQ793893.1	F	TCGTGGAGCGAATTTCCACA	106.20%	0.990
		R	GAGAACACGGACTCCCTTCG		
*myf5*	GU290227.1	F	TGAAGAAGGTGAACCACGCA	108.52%	0.992
		R	AGCTGCTTTCCATAGGCAGG		
*mstn1*	KM874826.1	F	CTGCCACAGGAGTCCAATGT	104.20%	0.991
		R	TGTCCATTCCCAAGTCCAGC		
*mtor*	JX854449	F	TCCCACTTTCCACCAACT	110.13%	0.986
		F	ACACCTCCACCTTCTCCA		
*mtmr8*	XM_051894113.1	F	TGATGGTTGGGACAGAACGG	102.00%	0.980
		R	GAGACACTTCCTTGGGGTCG		

## Data Availability

Data supporting the findings of this study are available in the article. Further inquiries should be addressed to the corresponding author.
